# The current situation of hereditary angioedema patients in Germany: results of an online survey

**DOI:** 10.3389/fmed.2023.1274397

**Published:** 2024-01-15

**Authors:** Markus Magerl, Inmaculada Martinez-Saguer, Lucia Schauf, Sven Pohl, Klaus Brendel

**Affiliations:** ^1^Institute of Allergology, Charité – Universitätsmedizin [Charité University Medical Department] Berlin and Fraunhofer-Institut für Translationale Medizin und Pharmakologie ITMP [Fraunhofer Institute for Translational Medicine and Pharmacology], Standort Allergologie und Immunologie [Allergology and Immunology], Berlin, Germany; ^2^Hämophilie-Zentrum Rhein Main [Hemophilia Center Rhein Main], Frankfurt, Germany; ^3^HAE Vereinigung e.V. [HAE Association], Aldenhoven, Germany; ^4^BioCryst Pharma Deutschland GmbH, Munich, Germany; ^5^Primus Consulting Group GmbH, Martinsried, Germany

**Keywords:** hereditary angioedema, burden of disease, long-term prophylaxis, quality of life, disease control, disease management, therapy satisfaction, oral therapy

## Abstract

**Introduction:**

Hereditary angioedema (HAE) is a rare hereditary disease with an estimated prevalence of approximately 1 in 50,000.

**Methods:**

An online survey was performed between January and June 2021 on a total of 99 HAE patients (with 92 of them aged 15 years and older and 7 of them being parents of patients under the age of 15 years). They were asked about their current situation, with a focus on the disease.

**Results:**

The survey results show that HAE has a strong influence on the patients’ quality of life. In particular, the anxiety and uncertainty of not knowing when a swelling attack will occur is considered burdensome by the patients. In addition, there can be physical problems during an attack (depending on its severity) that severely burden and limit patients in their everyday lives. Only one-third of the patients surveyed stated that no or only very minor physical limitations occurred during their most recent swelling attack. Almost three-quarters of all patients receive regular treatment at an HAE center. The patients are mostly satisfied with the therapy and particularly with long-term prophylactics (LTPs). When an LTP was used, the frequency and severity of the swelling attacks, and their duration, were significantly lower and/or shorter than when no LTP was used.

**Discussion:**

Despite the high level of satisfaction with their current medication, 62% of patients expressed a strong/very strong interest in an oral LTP. In the group of patients already using an LTP, 74% reported a strong/very strong interest in an oral medication for long-term prophylaxis. The simplicity and minimal time involved in LTP use are considered beneficial to patients’ quality of life.

## 1 Introduction

Hereditary angioedema (HAE) is a rare hereditary disease with an estimated prevalence of approximately 1 in 50,000 (1). There are approximately 1,700 patients with HAE in Germany. Being diagnosed with HAE is often a protracted process that takes years, sometimes decades. The swelling, particularly that of the face, extremities, genitals, and abdominal organs, occurs at irregular intervals and is mostly unpredictable. Based on the location, swelling episodes can be disfiguring, functionally impairing, or painful and affect patients as well as their families (2, 3).

By placing a stronger focus on research and medicine for rare diseases and the concurrent establishment of specialized centers, the options for both the diagnosis and the treatment of HAE have significantly improved over the past two decades (4, 5).

In collaboration with the self-help group HAE Vereinigung e.V.,^1^ a patient survey was conducted to collect current data about the lives of HAE patients in Germany, their management of the disease, and their satisfaction with the available treatment options for long-term prophylaxis.

## 2 Materials and methods

The survey was conducted online using a questionnaire containing a total of 77 questions. It was drafted by the primus consulting group and further developed by Markus Magerl, Inmaculada Martinez-Saguer, and Lucia Schauf. Following a pilot survey of five patients, the questionnaire was optimized, programmed for online use, and made available on the primus consulting group home page. HAE patients were made aware of the survey through both the home page and the Facebook page of HAE Vereinigung e.V. The original German language questionnaire was translated to English and included in the Supplementary material S1.

Prior to receiving a code to access the survey, potential participants registered for the survey and were verified by HAE Vereinigung e.V. for eligibility, which ensured that individuals not suffering from HAE could not participate. Survey entries occurred exclusively through the access code and all information provided by the participants was pseudonomized. Only the pseudonomized data were analyzed. Participants did not have to answer all questions.

Between January 27 and June 24, 2021, 92 patients aged 15 years and older, and seven parents of patients under the age of 15 years, participated in the survey (98 patients were from Germany and 1 patient was from Switzerland). Therefore, the survey sample size corresponds to 5–6% of the estimated total population of HAE patients in Germany. The average processing time for the questionnaire was 37 minutes, with a median of 32 minutes. Respondents provided answers to an average of 51.8 questions each. No group of questions was left unanswered as each group of questions contained at least one mandatory question, and all mandatory questions were answered by the participants. Participants received a gift card in the amount of 40 euros for completing the questionnaire. Seventy-three percent of survey participants were female, and the median age group was the 40–49 years group (Table 1). Almost two-thirds of patients (65%) had received a diagnosis of HAE with C1 inhibitor deficiency type I, followed by HAE with C1 inhibitor deficiency type II (17%), and HAE with normal C1 inhibitor (also called HAE type III; 5%). Thirteen percent of patients did not specify the type of HAE they were diagnosed with. Seventy-seven percent of the respondents reported that other family members suffer from HAE, with an average of 1.5 first-degree relatives and 2.1 relatives of higher degrees. One-quarter of patients surveyed stated that they do not currently use acute (on-demand) or long-term prophylactics (LTPs) for HAE. However, three-quarters of patients reported using on-demand medications, and half of all patients were treated with an LTP.

**Table 1:** Distribution of survey participants by age group and gender.

Age group, years	Total	Female	Male
<15	7 (7.1%)	6 (8.3%)	1 (3.7%)
15–17	2 (2.0%)	0 (0.0%)	2 (7.4%)
18–29	21 (21.2%)	14 (19.4%)	7 (25.9%)
30–39	16 (16.2%)	15 (20.8%)	1 (3.7%)
40–49	21 (21.2%)	16 (22.2%)	5 (18.5%)
50–59	18 (18.2%)	13 (18.1%)	5 (18.5%)
60–69	12 (12.1%)	7 (9.7%)	5 (18.5%)
≥70	2 (2.0%)	1 (1.4%)	1 (3.7%)
Total	99 (100.0%)	72 (100.0%)	27 (100.0%)

Using the Angioedema Control Test (AECT), patients’ disease status was assessed. The AECT is a validated tool for evaluating disease control in patients with recurrent angioedema. The test consists of four multiple choice questions (each with five answer options) to estimate a patient’s disease control over a recall period of 4 weeks or 3 months. Each of the five answer options is assigned a score of 0 to 4, resulting in a possible minimum score of 0 and a maximum score of 16. A total score ≥10 points indicates good disease control, whereas a therapy adjustment should be considered for patients with a score <10. In this patient survey, a recall period of 3 months was used, and patients who did not use on-demand or long-term prophylaxis in the last 3 months were not included in the analysis.

These survey results are only partially representative of the total HAE patient population in Germany because the characteristics of the target population (e.g., age, gender, geography) are unknown. Nevertheless, the results provide valuable insight into the current situation faced by HAE patients in Germany.

## 3 Results

### 3.1 Diagnosing HAE

The primary point of contact to establish a diagnosis was the general practitioner, who was seen by 83% of patients who answered the question (*n* = 57/69). On average, patients visited 2.9 specialists/institutions (e.g., medical clinic, HAE center) from the time of their first symptoms until their HAE diagnosis, which occurred predominantly at medical clinics (52%) or HAE centers (18%).

**Figure 1:**
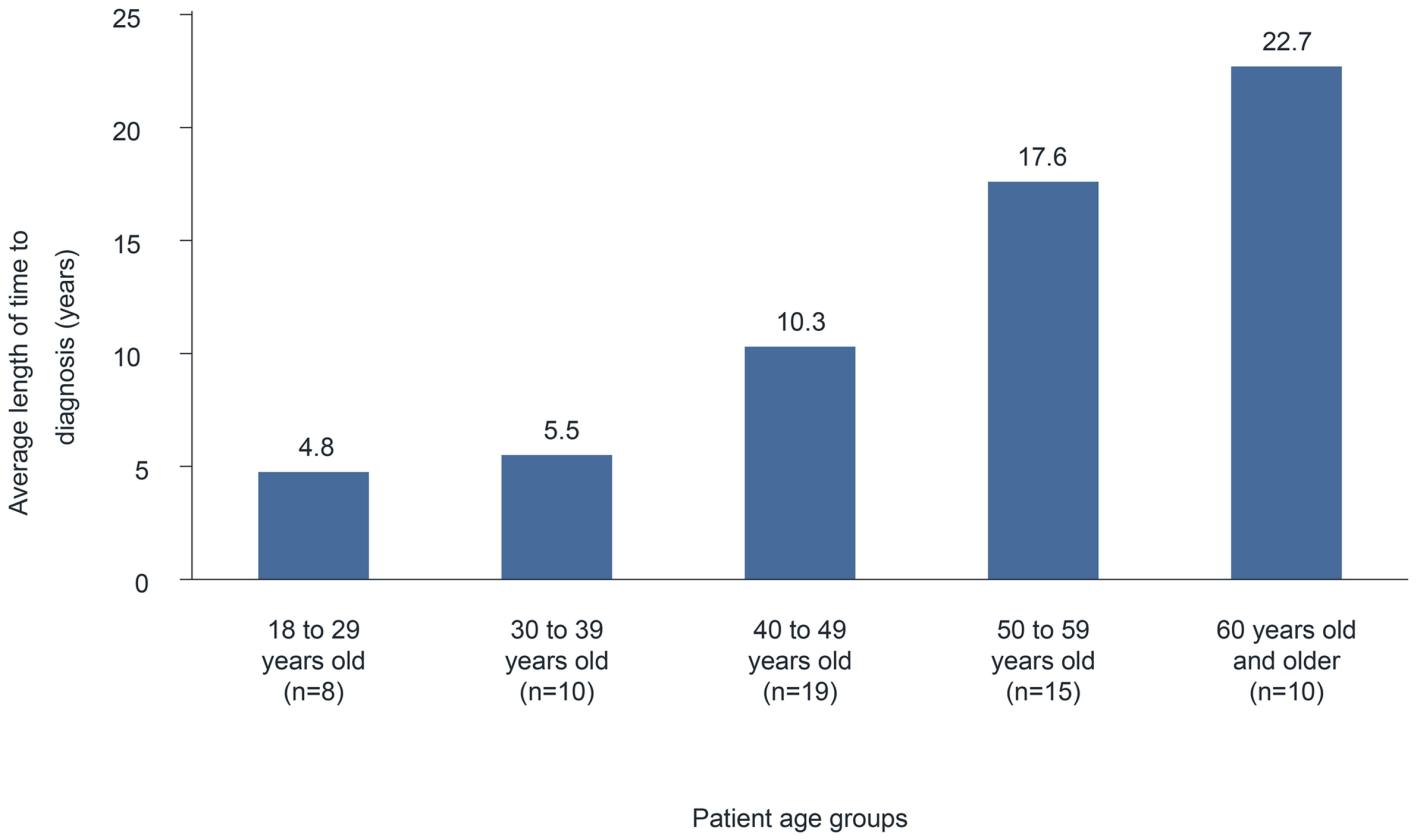
Average length of time from first symptoms to diagnosis according to patient age.

The average length of time (median) from the first appearance of symptoms until diagnosis was 12.6 (6) years (*n* = 62). A comparison between age groups suggests a correlation between the time to diagnosis and the patient’s age. Indeed, in younger patients (18–29 years), the average time to diagnosis was 4.8 years, which increased continuously with age (Figure 1) to 22.7 years in the ≥60 years age group. Similarly, in surveys of patients with HAE in Belgium and Mexico, participants reported a median time of 7 and 20 years, respectively, between HAE symptom onset and diagnosis (7, 8). Presumably, HAE has been diagnosed more rapidly in the past few years because of improved genetic testing options and the establishment of specialized centers (6, 9).

### 3.2 Disease burden and management

**Figure 2:**
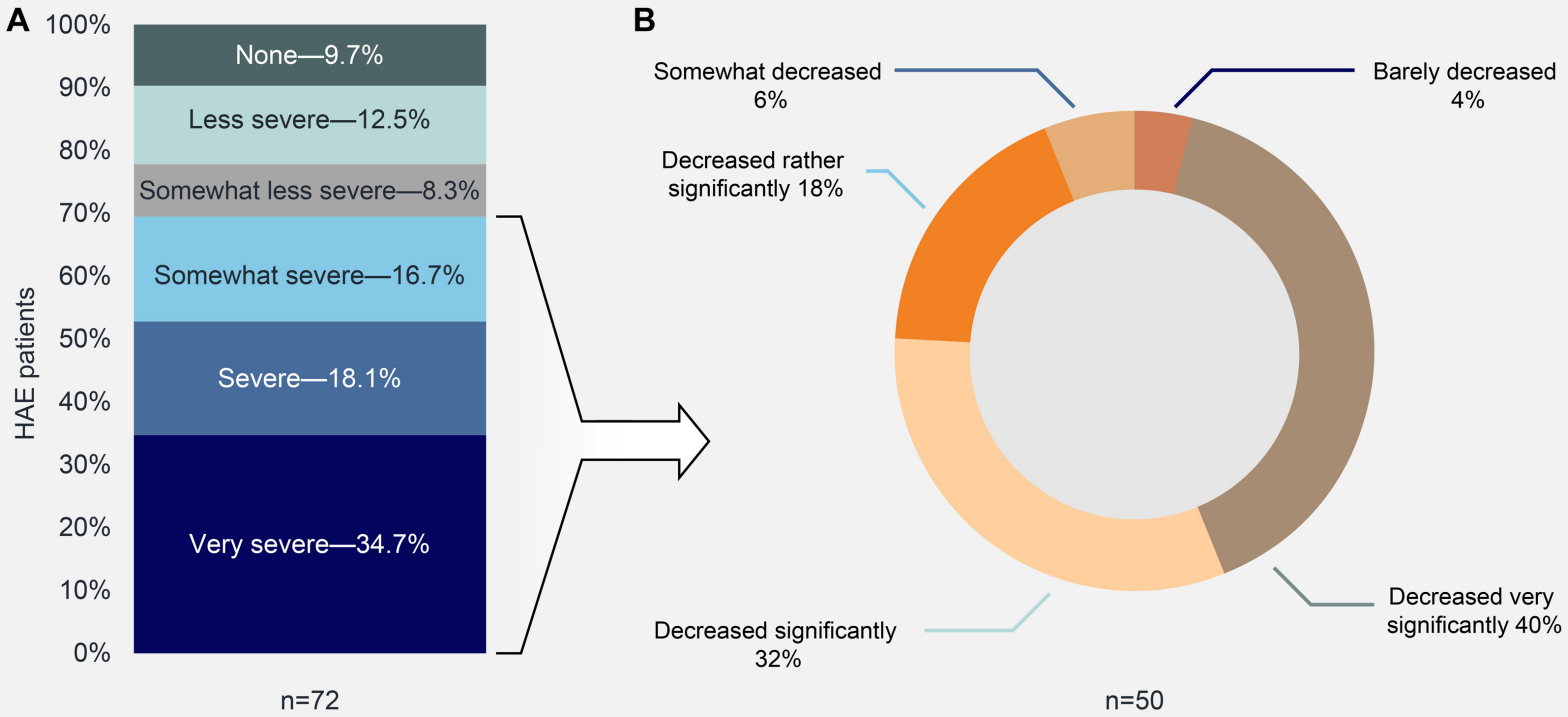
HAE interference with activities of daily life **(A)** before HAE diagnosis, and **(B)** after HAE diagnosis.

Approximately 70% of patients (*n* = 50/72) reported a “reported a “Somewhat severe” to “Very severe” interference interference of their HAE with their everyday life prior to their HAE diagnosis (Figure 2A). Following diagnosis, 90% of these patients (*n* = 45/50) reported a rather significant to very significant decrease in HAE interference with their daily life (Figure 2B). When asked about the most severe limitations due to their HAE, 32% of the 92 respondents stated that they currently do not have any limitations or have barely any limitations. Almost one-quarter of these patients attributed this to the use of prophylactic drugs.

According to the responses to the free-form questions, physical difficulties such as pain, limited mobility, and fatigue, as well as the anxiety/uncertainty related to the next attack, were equally important among the 63 patients who responded to the question (30% and 29% of patients, respectively). In addition, 16% of HAE patients (*n* = 10/63) described difficulties at work/school/preschool/daycare (e.g., sick leave, job loss) and 19% (*n* = 12/63) reported limitations during leisure time (particularly during sports and vacation activities). In isolated cases (6%, *n* = 4/63), patients also considered HAE prophylactic injections to be a constraint.

**Figure 3:**
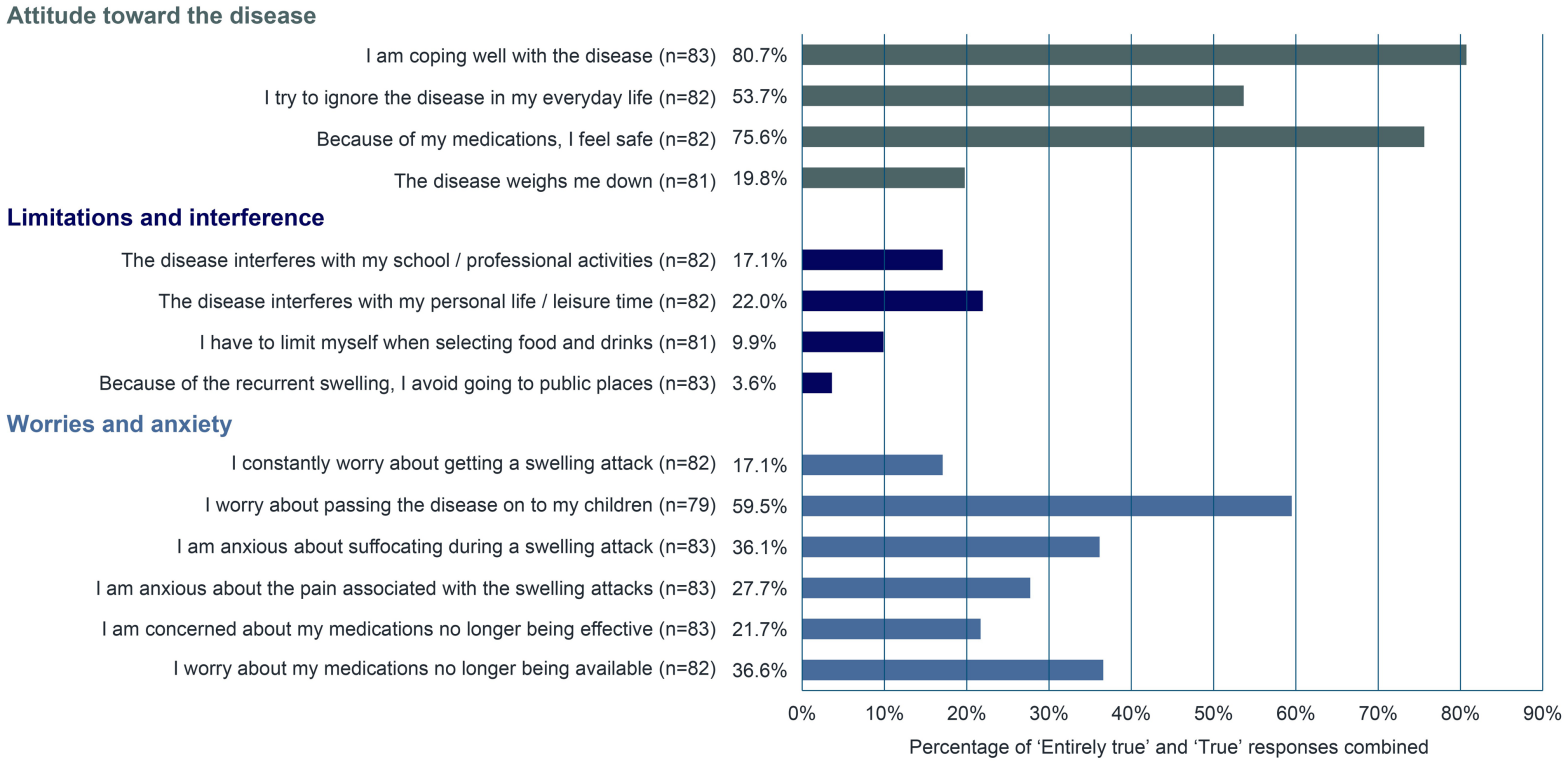
Disease management and attitude toward HAE.

Survey participants were also asked about their management of and attitude toward the disease using predetermined statements (Figure 3). Approximately 81% of respondents (*n* = 67/83) stated that they manage the disease well. Most patients (75.6%, *n* = 62/82) felt safe because of their medications. The patients’ biggest worry was passing the disease on to their children (59.5%, *n* = 47/79). The fear of suffocating during a swelling attack and the worry that medications may no longer be available were graded as “Entirely true” or “True” in approximately 36% of patients (*n* = 30/83 and *n* = 30/82, respectively). In a survey of 65 UK-based patients with HAE, approximately 64% (*n* = 7/11) of participants to a follow-up interview described throat swelling as their worst experience with HAE and their constant fear of pharyngeal attacks (10).

**Figure 4:**
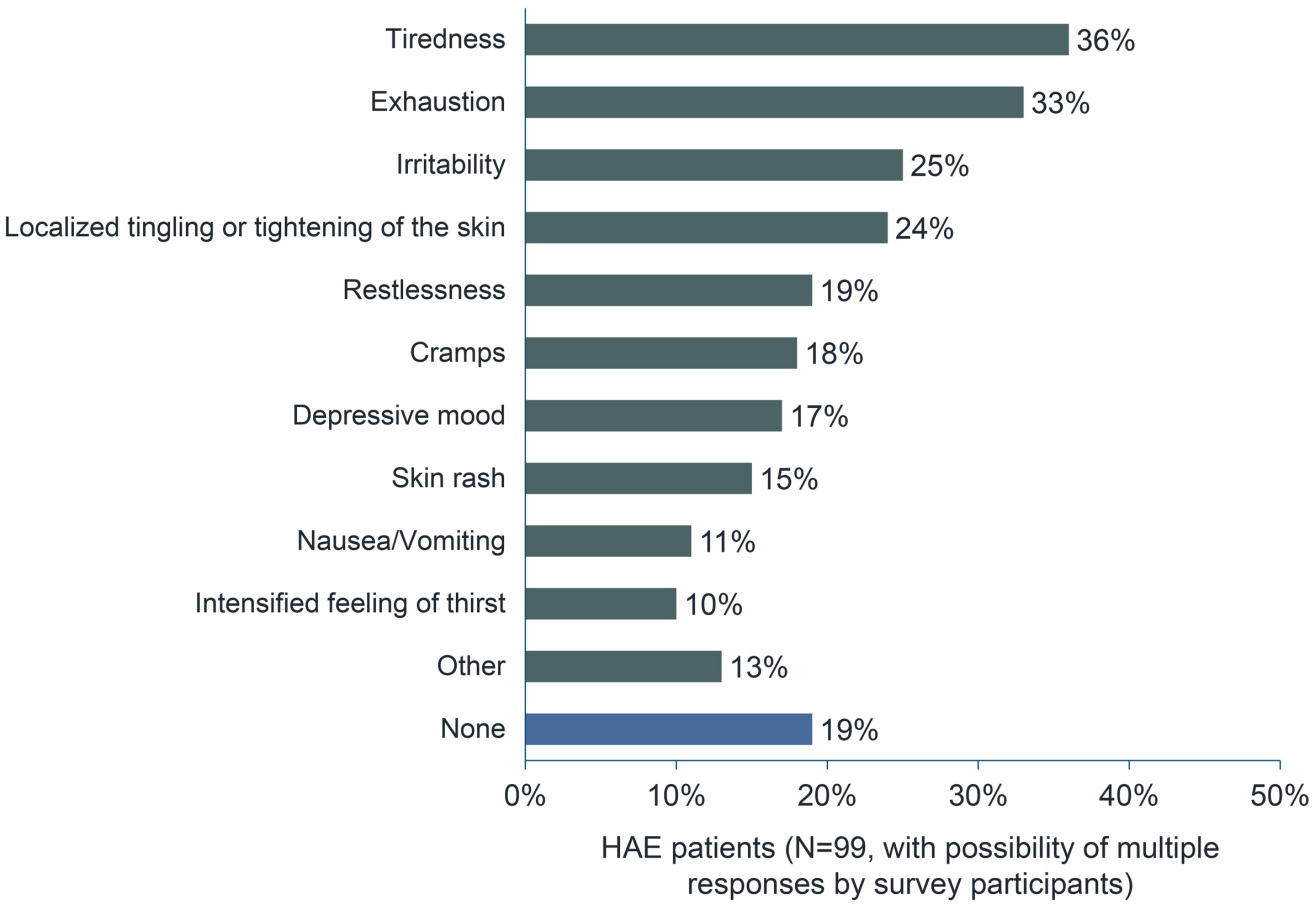
Symptoms preceding the most recent swelling attack.

Swelling attacks often begin with prodromal symptoms (11). More than 80% of respondents confirmed this with regard to their most recent episode. When asked which symptoms preceded their most recent swelling attack, 36% of respondents (*n* = 36/99) answered “Tiredness” and 33% (*n* = 33/99) answered “Exhaustion”. About one-quarter of the 99 respondents reported ‘Irritability’ and “Localized tingling or tightening of the skin” as prodromal symptoms (Figure 4).

**Figure 5:**
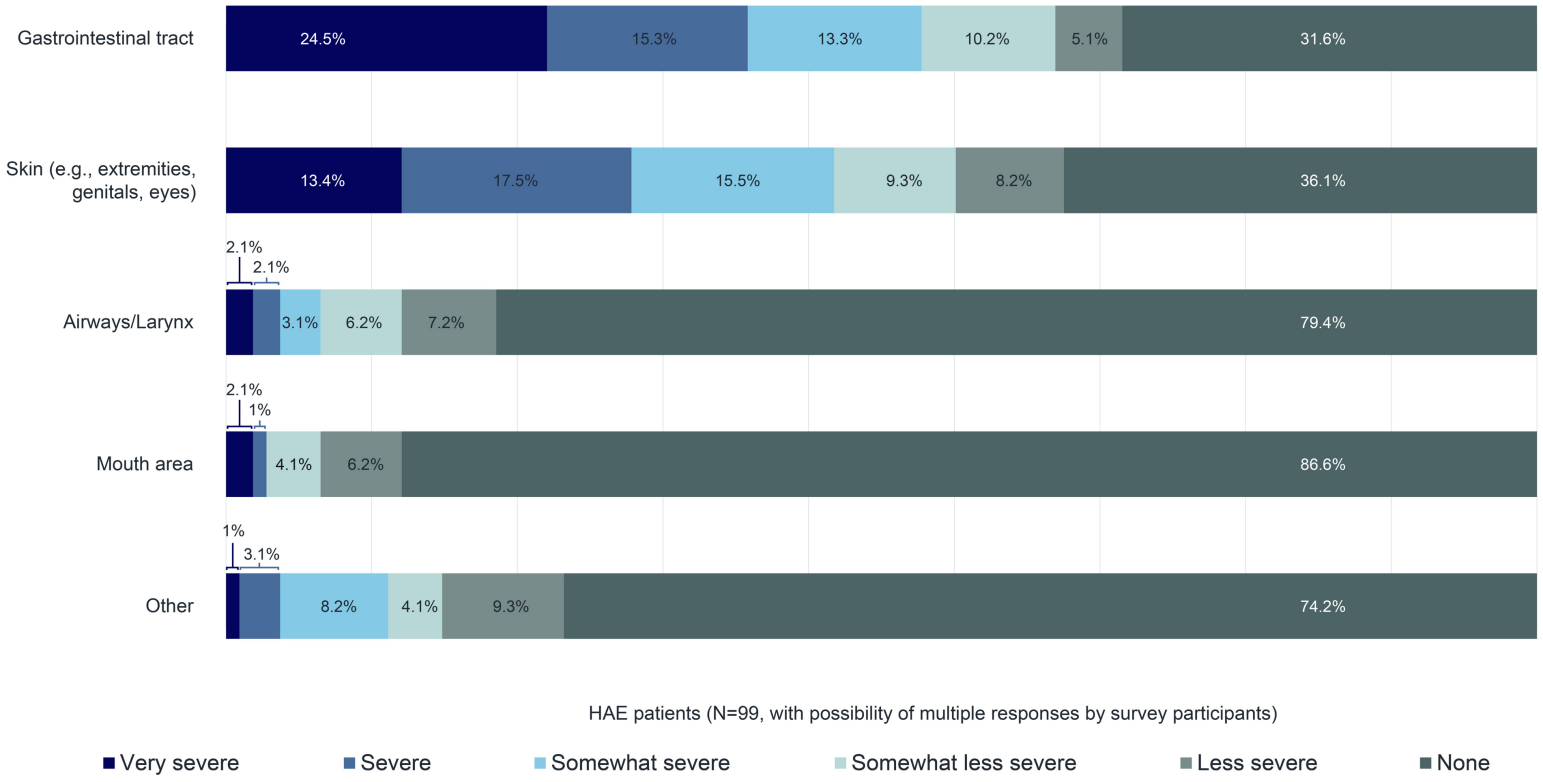
Body areas affected and swelling intensity during the most recent swelling attack.

Approximately 70% of the 99 patients reported that their last swelling attack affected their gastrointestinal tract. In 63.9% of patients, their skin (e.g., extremities, genitals, eyes) was involved. All other body areas were affected in less than one-quarter of patients (Figure 5). The prevalence of gastrointestinal and skin involvement compared with other body areas during HAE attacks has already been described in the literature (12). According to this survey, 53.1% of the 99 patients reported the intensity of the swelling affecting their gastrointestinal tract during their most recent swelling attack as either as either “Somewhat severe”, “Severe”, or “Very severe”, and 46.4% and 46.4% of patients reported the same intensity for their skin (Figure 5).

Thirty-six percent of all the patients surveyed did not have a swelling attack during the previous 3 months, and 20% had at least seven attacks (median of all patients: two swelling attacks in the last 3 months). On average, the length of the most recent swelling attack was 34 hours (median: 24 hours, *n* = 97).

### 3.3 Medical care for HAE patients

Seventy-one percent of patients (*n* = 69/97) stated that they receive regular medical care for their HAE disease at an HAE center, and an additional 13% receive treatment at a clinic. Among specialist doctors, only general practitioners have a degree of involvement, with 31% of HAE patients (*n* = 30/97) receiving regular HAE care from their general practitioner. Allergists (7%) and dermatologists (7%), as well as ear, nose, and throat specialists and pediatricians (both 3%), play a more minor role when it comes to the care of HAE patients, unless they are based in HAE centers. This mirrors the results from surveys of HAE patients in other parts of the world (7, 8, 10) highlighting the importance of awareness and knowledge of HAE management guidelines in general practitioners.

Based on 77 respondents, half of HAE patients who receive regular care at an HAE center or clinic have to travel more than 200 km to get there. This is also reflected in the effort involved to commute to and from the treatment facility: on a scale of 1 (very low) to 6 (very high), survey participants rated that effort rated that effort 4.4, relatively high. Patients travel to and from a center or clinic predominantly by car (63%, *n* = 47/75). The wait time at the center or the clinic was deemed “Moderately low” to “Very low” by 70% of respondents, whereas 30% of respondents considered the wait time to be “Moderately high” or “Very high”. Overall, patients rated the medical/therapeutic support for their disease as “good” or “very good” (80%, *n* = 79/99). The medical/therapeutic support was rated as “Inadequate” or “Unsatisfactory” only in rare instances (three patients: one patient receives treatment from their general practitioner; one patient is treated at an HAE center; and one patient did not specify a medical group but commented “Have not found a doctor near my home”).

A little more than two-thirds of HAE patients (68%, *n* = 63/93) obtain their prescribed medication at a local pharmacy, whereas 19% use an online service or pharmacy delivery. In rare cases, patients covered by statutory health insurance stated that their supplementary payments at pharmacies are very high (e.g., 200 euros for 20 ampoules). Since these amounts far exceed the supplementary payment for medications determined by German law (maximum of 10 euros per medication), there seems to be a need for education about the supplementary payment limits, both for pharmacies (e.g., through the manufacturers) and for patients.

### 3.4 HAE therapy

According to the survey, 88% of patients (*n* = 87/99) attempted to manage their most recent swelling attack with HAE medications and other measures such as heated pillows, analgesia, and Iberogast^®^. Half of those who did not treat their attack stated that the swelling affected their skin and/or gastrointestinal tract “Severely” to “Very severely”. A large proportion of patients (84%, *n* = 83/99) reported taking an HAE medication during their most recent swelling attack. Almost half of these patients (45%) either self-administered or received an injection of Berinert^®^. Far fewer patients used Firazyr^®^ (22%) or Cinryze^®^ (12%). Fifty percent of those who initiated treatment during their most recent swelling attack did so within 1 h of the onset of symptoms (*n* = 44/88). Very few patients (6%, *n* = 5/88) did not take any action for more than 6 h. It was not possible to establish a correlation between time to treatment and the type of medication. Despite early treatment (median of 1.0 h) of their most recent swelling attack, the average (median) duration of symptoms during the attack was 34.0 (24.0) hours (*n* = 97).

Overall, patients are satisfied with their HAE therapy. On a scale of 1 (very satisfied) to 6 (very dissatisfied), an average score of 1.7 was reported across all HAE medications. Satisfaction with on-demand medication and LTPs was aligned with the overall satisfaction. However, androgen therapy and tranexamic acid, which are rarely used today, reached a score of approximately 3 (but with very few mentions).

**Figure 6:**
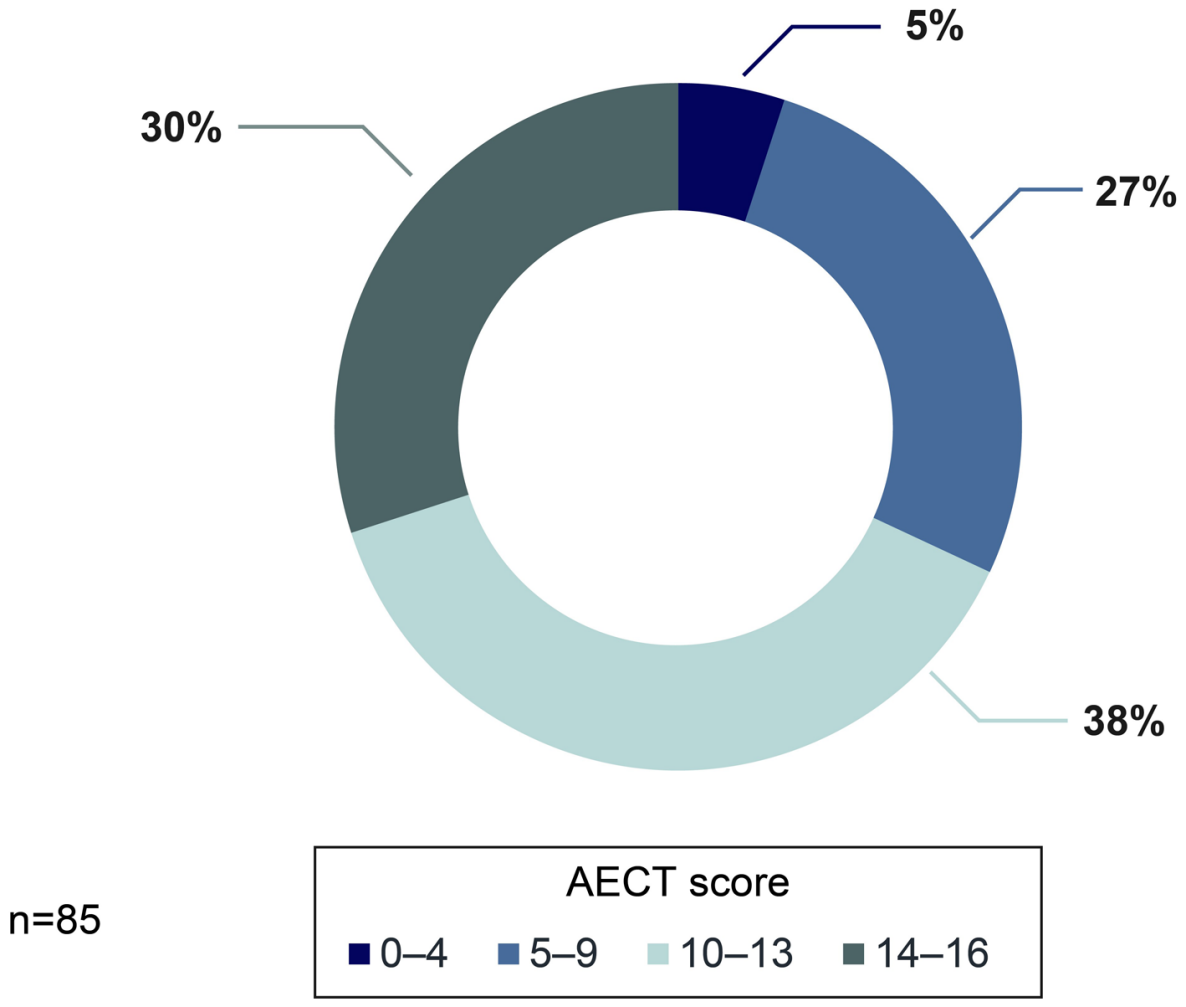
Distribution of HAE patients according to AECT score.

Results from the AECT showed that 68% of patients (*n* = 58/85) reached a score of at least 10 points, and 19% reached the maximum score of 16 points. However, one-third of patients scored <10, suggesting that disease control in these patients is suboptimal (Figure 6).

### 3.5 Long-term prophylaxis for HAE

**Figure 7:**
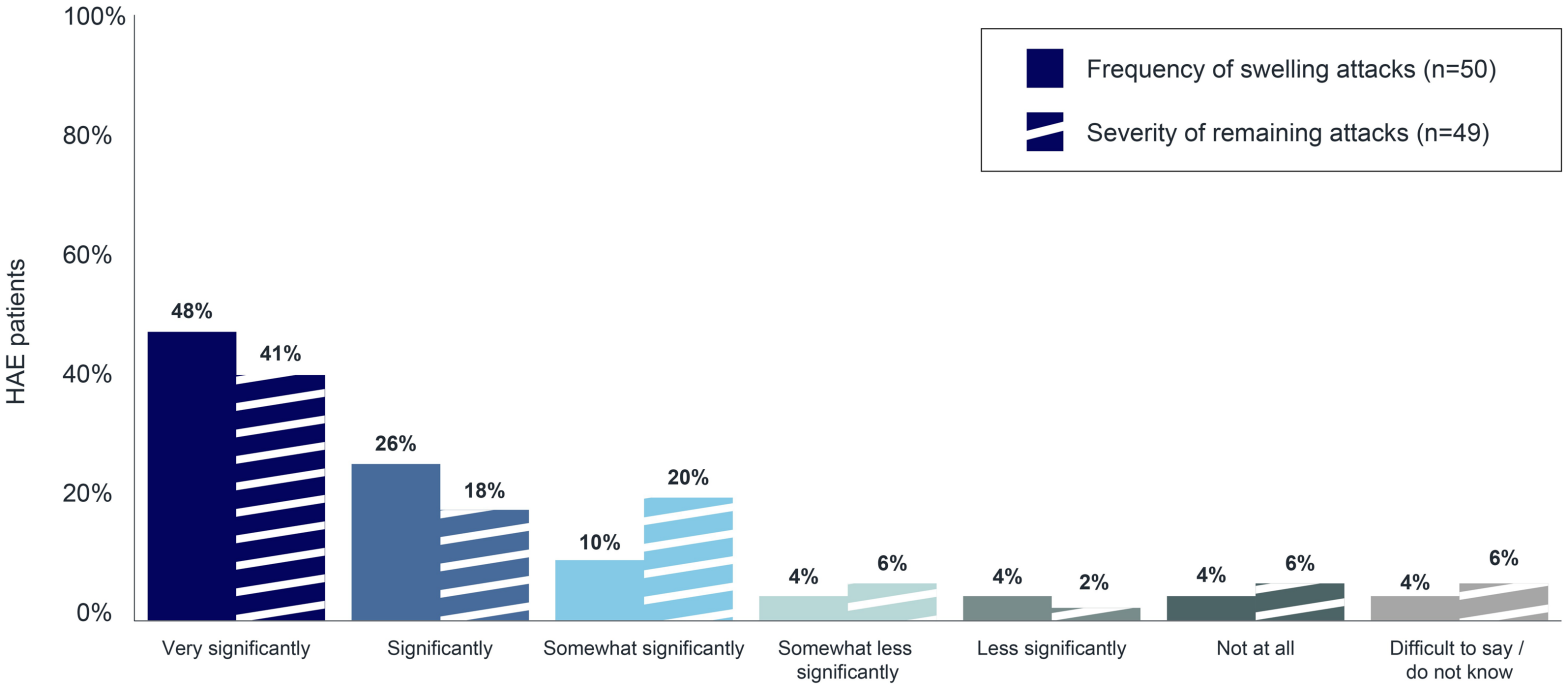
Decrease in frequency and severity of swelling attacks associated with long-term prophylaxis.

The goal of long-term prophylaxis in patients with HAE is to reduce the number and/or severity of attacks, to (among other things) improve disease control and patients’ quality of life (13). To better understand the extent of the positive impact that long-term prophylaxis has on HAE attacks, patients were asked to rate the degree of change in the frequency and severity of their swelling attacks since they began taking an LTP. Seventy-four percent (*n* = 37/50) and 59% (*n* = 29/49) of patients on long-term prophylaxis reported experiencing a very significant or significant decrease in the frequency and in the severity of their swelling attacks, respectively (Figure 7).

**Figure 8:**
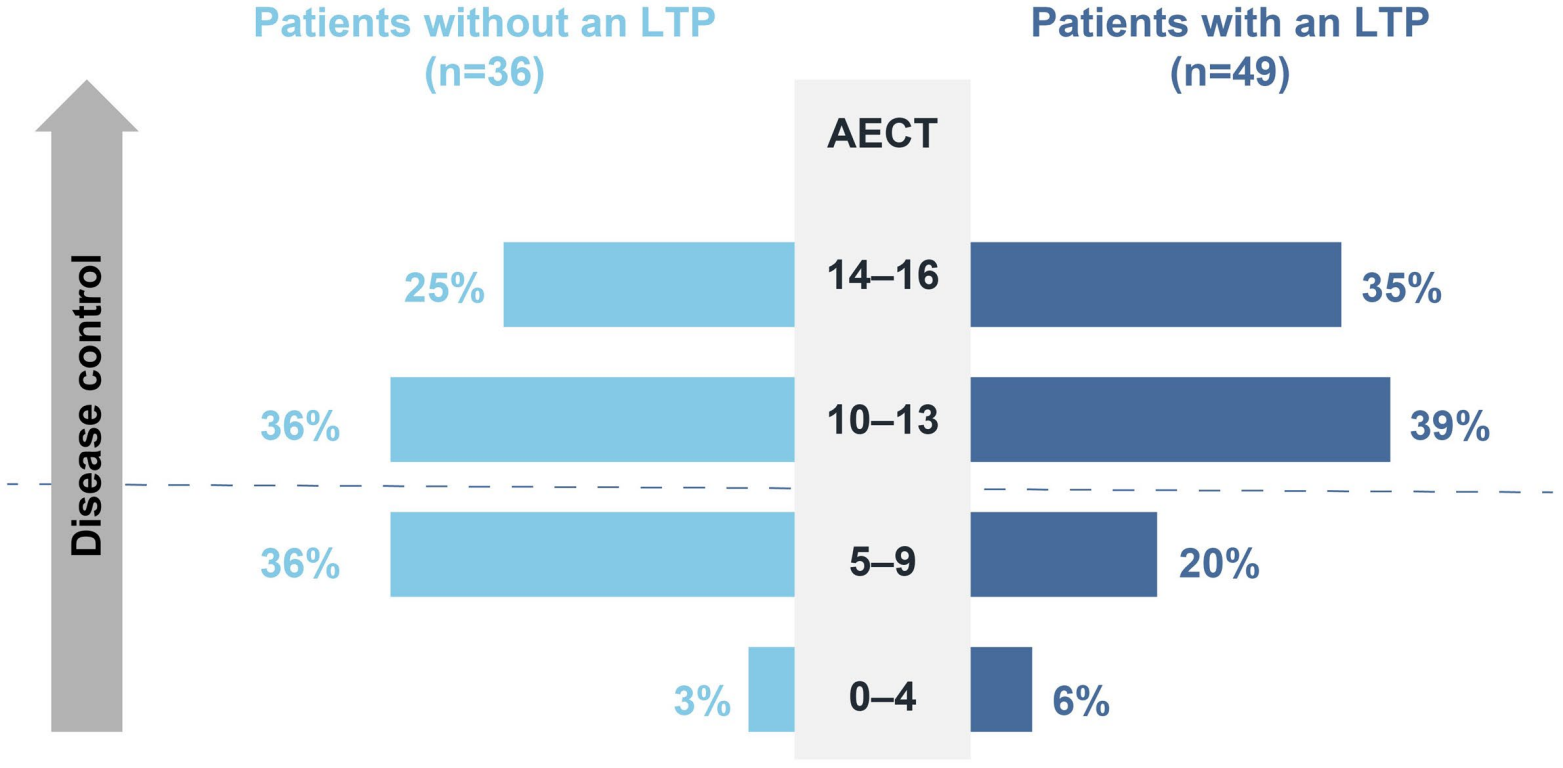
AECT score (3-month recall period).

A comparison of AECT scores between HAE patients on long-term prophylaxis and those who do not use long-term prophylaxis demonstrated the positive impact of LTPs on disease control. Indeed, Almost 74% of patients on long-term prophylaxis (*n* = 36/49) reached an AECT score of ≥10 points, whereas only 61% of patients who do not use long-term prophylaxis (*n* = 22/36) obtained the same result (Figure 8). Accordingly, 82% of patients on long-term prophylaxis (*n* = 41/50) are “Satisfied” or “Very satisfied” with their LTP, and 74% reported a “Significant” to “Very significant” improvement in their quality of life as a result of their LTP (*n* = 37/50).

**Figure 9:**
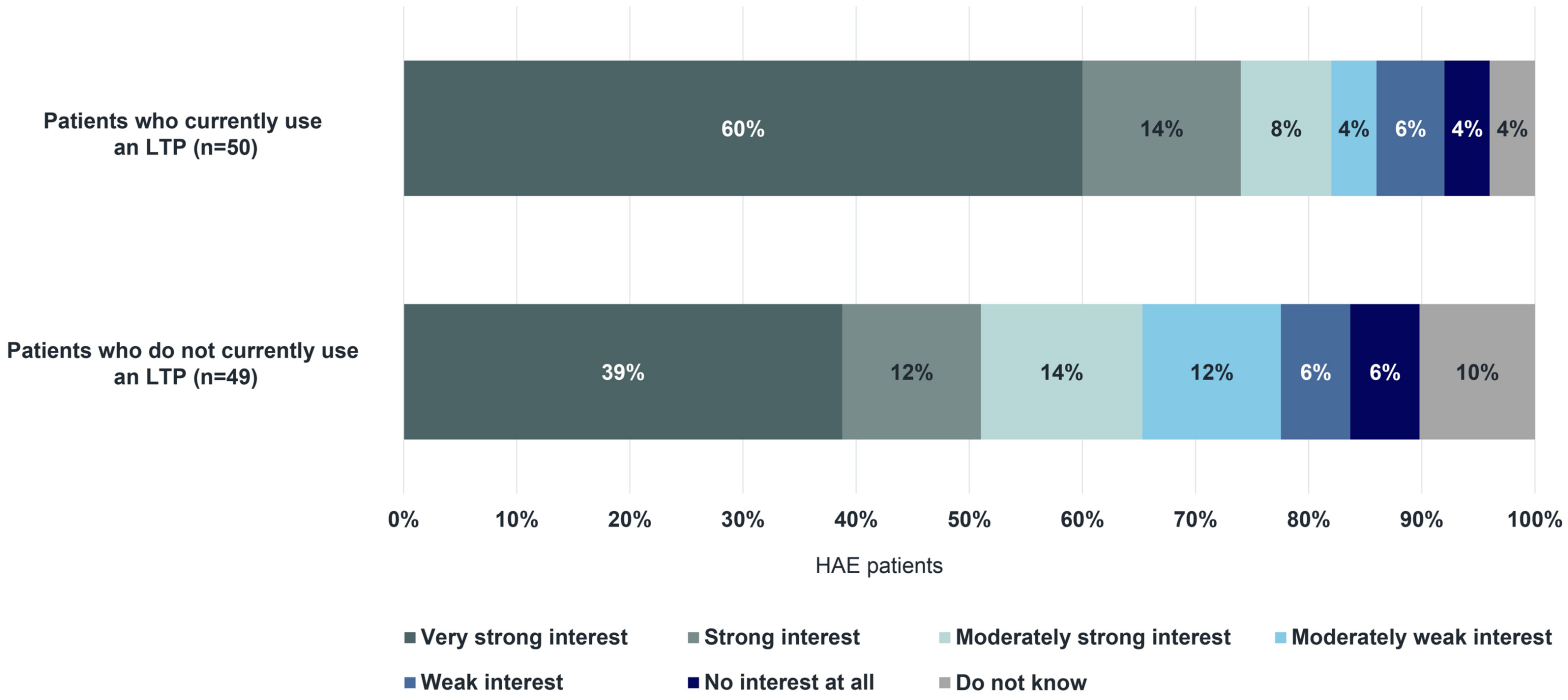
Interest in a tablet for long-term prophylaxis.

Almost all HAE patients surveyed (96%, *n* = 95/99), including some who do not currently use an LTP, indicated that long-term prophylaxis has advantages. A large majority of patients mentioned the reduction in the frequency of their swelling attacks (83%) and the improvement in their quality of life (73%). Increased flexibility in their professional and personal lives, and a decrease in worries about swelling attacks were advantages reported by 67% and 66% of patients, respectively. This was closely followed by the reduction in the severity of attacks (61%). Of the 50 patients on long-term prophylaxis who responded and the 49 patients not currently using long-term prophylaxis who responded, two-thirds also reported the disadvantages of currently available treatment: breakthrough attacks (36% with an LTP; 18% without an LTP), forgetting to use it regularly (22% with an LTP; 22% without an LTP), and the effort involved in preparing for treatment (18% with an LTP; 18% without an LTP). Among both groups, the majority indicated a “Very strong interest” to “Strong interest” in an oral formulation for long-term prophylaxis for HAE (Figure 9). Surveyed patients rated the ease of administration (90%) and the short time needed to take an oral LTP (93%) as “True” and “Entirely true”. Furthermore, they mentioned that a physician’s recommendation for the selection of an oral formulation is important (84% indicated this to be “True” or “Entirely true”). Lastly, 68% of patients surveyed (*n* = 67/99) reported that it is “Likely”, “Very likely”, or “Definite” that they will try an oral option for long-term prophylaxis for HAE.

### 3.6 Contact with other patients and use of HAE-specific services

Almost half of all 99 survey participants (47%) are in contact with other HAE patients even outside their own family. According to one-third of these patients, these contacts occur from once to several times per quarter. Eighty-four percent of HAE patients surveyed (*n* = 83/99) used a patient diary/swelling calendar during the last 12 months, whereas 60% used an emergency card (which informs healthcare professionals about their disorder). Surveyed patients also turn to services that provide information about the disease in general (38%), delivery service for medications (33%), and HAE patient experiences/stories (25%), but to a lower extent. Additional services offered, such as the “HAE expert search on the internet” (14%), “physician’s video/phone consultation hours” (13%), and “therapy counselor” (9%), were rarely used during the last 12 months by HAE patients surveyed. On average, the support received from the professional and educational environment is considered ‘adequate’ by survey participants. More specifically, patients asked for information they could use to inform their employer, teacher/professor, or physician/healthcare professional when they have an HAE swelling attack. Some HAE patients are also interested in information about new therapy options and ongoing studies in a newsletter format.

## 4 Conclusion

This survey of HAE patients in Germany provides valuable insight into the everyday lives of these patients, their disease management, and their satisfaction with the current treatment options. The results indicate that the time between the onset of HAE symptoms and diagnosis has significantly declined during the past few decades, which can likely be associated with physicians’ greater awareness of rare diseases, access to genetic testing, and the availability of specialized HAE centers. Diagnosis and treatment can significantly reduce the negative impact of HAE on everyday life. LTPs lead to better disease control compared with other therapies and reduce the frequency and severity of swelling attacks. Patients on long-term prophylaxis are highly satisfied with their treatment and simultaneously express a great deal of interest for an oral formulation for long-term prophylaxis for HAE.

## Data availability statement

The raw data supporting the conclusions of this article will be made available by the authors, without undue reservation.

## Ethics statement

Ethical review and approval was not required for the study on human participants in accordance with the local legislation and institutional requirements. Written informed consent from the participants was not required to participate in this study in accordance with the national legislation and the institutional requirements.

## Author contributions

MM: Conceptualization, Methodology, Writing – original draft, Writing – review & editing. IM-S: Conceptualization, Methodology, Writing – review & editing. LS: Conceptualization, Methodology, Writing – review & editing. SP: Conceptualization, Methodology, Writing – original draft, Writing – review & editing. KB: Conceptualization, Methodology, Writing – original draft, Writing – review & editing.

## Footnotes

^1^ www.hae-online.de
