# Supplementary material for: The current situation of hereditary angioedema patients in Germany: results of an online survey
Source: Front Med (Lausanne). 2024 Jan 15;10:1274397. doi: 10.3389/fmed.2023.1274397 (PMC10822932; doi:10.3389/fmed.2023.1274397)
Supplement: Supplementary file 1 [file Data_Sheet_1.docx]

**Supplement**

**Online questionnaire for patients with hereditary angioedema in Germany**

***This questionnaire was developed and administered to survey participants in German. It has been translated to English for inclusion in this article.***

* Mandatory questions

***Section A: Diagnosis***

*** Question A0:** At what age were you diagnosed with hereditary angioedema? *(Please check)*

- Before my 6^th^ birthday 🡪 Continue to **question A4**
- After my 6^th^ birthday 🡪 Continue to **question A1**

**Question A1:** Approximately when did you experience the first HAE symptoms? *(Please enter month and year – if month not known, please enter 00)*

| Month |  |  | Year |  |
| --- | --- | --- | --- | --- |

**Question A2*:** Had you already received any indications of the suspected diagnosis from the family environment before the diagnosis was made? *(only one selection possible)*

| Yes | Partially | No | Don't know |
| --- | --- | --- | --- |
| 🌕 | 🌕 | 🌕 | 🌕 |

**Question A3:** Which (specialist) physicians did you visit after the symptoms occurred? (*Please check – multiple answers are possible)*

- GP
- Dermatologist
- Allergist
- Pediatrician
- Gastroenterologist
- Pulmonologist
- Otolaryngologist
- HAE Centre
- Clinic/Hospital
- Other, please specify:

*** Question A4:** Where was HAE ultimately diagnosed? *(Please check – only one entry possible)*

- GP
- HAE Centre
- Clinic/Hospital (no HAE centre)
- Other, please specify:

**Question A5:** When were you diagnosed with HAE? *(Please enter month and year – if month not known, please enter 00)*

| Month |  |  | Year |  |
| --- | --- | --- | --- | --- |

*** Question A6:** What type of HAE were you diagnosed with? *(Please check – only one entry possible)*

- Type I (too little C1 inhibitor is produced)
- Type II (C1 inhibitor not working properly)
- Type III (no C1 inhibitor deficiency)
- Don't know

**Question A7.1:** Have other people in your family been diagnosed with HAE?

- Yes 🡪 Continue to **question A7.2**
- No 🡪 Continue to **question B1**
- Don’t know 🡪 Continue to **question B1**

| **Question A7.2:** | Please indicate the number of people in your family who have been diagnosed with HAE. | |
| --- | --- | --- |
|  | In how many first-degree relatives (parents, children)? |  |
|  | | |
|  | In how many higher-degree relatives (e.g., siblings, grandparents, aunts, uncles, grandchildren, cousins, nephews, nieces)? |  |
|  |  |  |
|  | Continue to **question B1** |  |

***Section B: Quality of life***

***If respondents indicated in question A0 that their diagnosis was made before the 6th birthday, questions B1 and B2 were not displayed and participants could not respond to those questions***

**Question B1:** Thinking about the time before you were diagnosed, how much do you think your daily life was impaired during that time, from today's perspective? *(Please check)*

My impairment before diagnosis was:

| Very severe | Severe | Somewhat severe | Somewhat less severe | Less severe | None |
| --- | --- | --- | --- | --- | --- |
| 🌕 | 🌕 | 🌕 | 🌕 | 🌕 | 🌕 |

**Question B2:** If you compare the impairments at the time with your situation today, how would you describe the change? *(Please check – only one entry possible)*

My impairments have:

| Decreased very significantly | Decreased significantly | Decreased rather significantly | Somewhat decreased | Barely decreased | Not decreased |
| --- | --- | --- | --- | --- | --- |
| 🌕 | 🌕 | 🌕 | 🌕 | 🌕 | 🌕 |

**Question B3:** What are currently the most severe limitations of the disease for you? *(Please enter)*

**Question B4:** How would you describe your current handling of your HAE condition / attitude towards your HAE condition?

For each statement on the list, please check how well the statement applies to you:

|  | Applies fully | Applies | Somewhat applies | Somewhat does not apply | Does not apply | Does not apply at all |
| --- | --- | --- | --- | --- | --- | --- |
| I am coping well with the disease | 🌕 | 🌕 | 🌕 | 🌕 | 🌕 | 🌕 |
| I try to ignore the disease in my daily life | 🌕 | 🌕 | 🌕 | 🌕 | 🌕 | 🌕 |
| I feel very safe because of my medication | 🌕 | 🌕 | 🌕 | 🌕 | 🌕 | 🌕 |
| The disease weighs me down | 🌕 | 🌕 | 🌕 | 🌕 | 🌕 | 🌕 |
| I am constantly worried about having a swelling attack | 🌕 | 🌕 | 🌕 | 🌕 | 🌕 | 🌕 |
| The disease interferes with my  school / professional activities | 🌕 | 🌕 | 🌕 | 🌕 | 🌕 | 🌕 |
| The disease interferes with my personal life / leisure time | 🌕 | 🌕 | 🌕 | 🌕 | 🌕 | 🌕 |
| I have to limit myself a lot when selecting food and drinks | 🌕 | 🌕 | 🌕 | 🌕 | 🌕 | 🌕 |
| I am concerned about passing the disease on to my children | 🌕 | 🌕 | 🌕 | 🌕 | 🌕 | 🌕 |
| Because of the recurring swelling, I avoid going to public places | 🌕 | 🌕 | 🌕 | 🌕 | 🌕 | 🌕 |
| I am anxious about suffocating during an HAE attack | 🌕 | 🌕 | 🌕 | 🌕 | 🌕 | 🌕 |
| I am afraid of the pain associated with HAE attacks | 🌕 | 🌕 | 🌕 | 🌕 | 🌕 | 🌕 |
| I am concerned that my medication will no longer work | 🌕 | 🌕 | 🌕 | 🌕 | 🌕 | 🌕 |
| I am worried that my medication will no longer be available | 🌕 | 🌕 | 🌕 | 🌕 | 🌕 | 🌕 |

**Question B5:** Approximately how often have you consulted a physician / different physicians because of HAE in the past 6 months? *(Please check)*

|  | 0x | 1x | 2x | 3x | 4x | 5x | 6x | More than 6x |
| --- | --- | --- | --- | --- | --- | --- | --- | --- |
| GP | 🌕 | 🌕 | 🌕 | 🌕 | 🌕 | 🌕 | 🌕 | 🌕 |
| HAE Centre | 🌕 | 🌕 | 🌕 | 🌕 | 🌕 | 🌕 | 🌕 | 🌕 |
| Specialist  (non-HAE centre) | 🌕 | 🌕 | 🌕 | 🌕 | 🌕 | 🌕 | 🌕 | 🌕 |

*** Question B6:** How often have you had angioedema in the past 3 months? *(Please select one of the following answers)*

- Very often
- Often
- Occasionally
- Rarely
- Not at all

*** Question B6a:** How much has your quality of life been affected by angioedema in the last 3 months?
*(Please select one of the following answers)*

- Very severely
- Severely
- Moderately
- Barely
- Not at all

*** Question B6b:** How much has the unpredictability of angioedema burdened you in the last 3 months?
*(Please select one of the following answers)*

- Very severely
- Moderately
- Barely
- Not at all

*** Question B6c:** How well has your angioedema been controlled by your therapy in the last 3 months?
*(Please select one of the following answers)*

- Not at all
- Barely
- Moderately
- Well
- Very well
- I currently have no therapy

**Question B7:** Thinking about the last 3 months, how many attacks did you have in the periods mentioned? *(Please enter)*

| In the last 4 weeks |  |
| --- | --- |
|  |  |
| In the last 3 months |  |

**Question B8:** Thinking about your last attack, were there any symptoms that preceded the actual swelling attack?
*(Please select all applicable answers)*

- No
- Yes, fatigue
- Yes, exhaustion
- Yes, increased thirst
- Yes, irritability
- Yes, depressive mood
- Yes, localised tingling or tightening of the skin before the onset of openly visible swelling
- Yes, skin rash
- Yes, restlessness
- Yes, joint pain
- Yes, nausea / vomiting
- Yes, cramps
- Yes, other, please specify:

**Question B9:** Thinking about your last attack (swelling), what body regions were affected and how severely?
*(Please check for each body region)*

|  | Very severely | Severely | Somewhat  severely | Somewhat  less severely | Less severely | Not at all |
| --- | --- | --- | --- | --- | --- | --- |
| Skin (e.g., extremities, genitals, eye) | 🌕 | 🌕 | 🌕 | 🌕 | 🌕 | 🌕 |
| Gastrointestinal tract | 🌕 | 🌕 | 🌕 | 🌕 | 🌕 | 🌕 |
| Respiratory tract / larynx (with difficulty breathing) | 🌕 | 🌕 | 🌕 | 🌕 | 🌕 | 🌕 |
| Mouth area (e.g., tongue) | 🌕 | 🌕 | 🌕 | 🌕 | 🌕 | 🌕 |
| Other | 🌕 | 🌕 | 🌕 | 🌕 | 🌕 | 🌕 |

**Question B10:** And approximately how long did the last attack (swelling) last? You have the option of entering the duration of the last attack **in either hours or days**. *(Please enter the appropriate value for either hours or days; you can also indicate 0.5 for half days, for example)*

| Hours: |  |
| --- | --- |
|  |  |
| Days: |  |

**Question B11:** Thinking about your last swelling attack, what treatment measures did you take? *(Please specify)*

**Question B11a:** Approximately how many hours after the onset of the symptoms did you start the treatment measures?

| Hours: |  |
| --- | --- |

*** Question B12.1:** Are you in contact with other HAE patients (outside your family)?

- Yes 🡪Continue to **question B12.2**
- No 🡪Continue to **question B13**

**Question B12.2:** Approximately how frequent are these contacts? *(Please select one of the following answers)*

- More frequently than 1x per month
- Approximately 1x per month
- Approximately 1-2x per quarter
- Approximately 1x in six months
- Approximately 1x per year
- Less frequently than 1x per year

**Question B13:** How well do you feel supported in terms of your HAE condition? *(Please check)*

|  | Very well | Well | Satisfactorily | Sufficiently | Inadequately | Insufficiently | Not applicable |
| --- | --- | --- | --- | --- | --- | --- | --- |
| Medically / therapeutically | 🌕 | 🌕 | 🌕 | 🌕 | 🌕 | 🌕 | 🌕 |
| In the school / professional area / environment | 🌕 | 🌕 | 🌕 | 🌕 | 🌕 | 🌕 | 🌕 |
| In the private area / environment | 🌕 | 🌕 | 🌕 | 🌕 | 🌕 | 🌕 | 🌕 |
| By patient association / self-help group | 🌕 | 🌕 | 🌕 | 🌕 | 🌕 | 🌕 | 🌕 |

| By patient services (from drug manufacturers, e.g., information material, emergency passports) | 🌕 | 🌕 | 🌕 | 🌕 | 🌕 | 🌕 | 🌕 |
| --- | --- | --- | --- | --- | --- | --- | --- |
| By homecare (home visits by nurse) | 🌕 | 🌕 | 🌕 | 🌕 | 🌕 | 🌕 | 🌕 |
| Other, please specify: | 🌕 | 🌕 | 🌕 | 🌕 | 🌕 | 🌕 | 🌕 |

**Question B14:** Is there any other support that would be additionally helpful for you (e.g., educational material, service)? *(Please check)*

- No
- Yes, which? *(Please specify)*

***Section C: Medication***

*** Question C00:** Are you currently taking any medication for treating attacks / on-demand medication for HAE? *(Please check as appropriate, only one selection is possible)*

- Yes 🡪Continue to **question C01.1a**
- No 🡪Continue to **question C06**

*** Question C01.1a:** What medication do you currently use to **treat your HAE attacks / for on-demand treatment?** (*Please check only the medication(s) you are* ***currently*** *taking for treating attacks / on-demand treatment)*

- Berinert® 500 / 1500
- Berinert® 2000 / 3000 (SC)
- Cinryze®
- Ruconest®
- Firazyr®
- Takhzyro®
- Androgens (e.g., danazol, stanozolol, oxandrolone)
- Tranexamic acid (e.g., Exacyl®)
- None of the above

*** Question C01.1b:** What medications do you currently use for **long-term prophylaxis** of HAE? *(Please check only the medication(s) you are* ***currently*** *taking for long-term prophylaxis)*

- Berinert® 500 / 1500
- Berinert® 2000 / 3000 (SC)
- Cinryze®
- Ruconest®
- Firazyr®
- Takhzyro®
- Androgens (e.g., danazol, stanozolol, oxandrolone)
- Tranexamic acid (e.g. Exacyl®)
- None of the above

*** Question C01.1c:** Which medications do you currently use for **short-term prophylaxis (only before surgeries or similar)** of HAE? *(Please check only the medication(s) you are* ***currently*** *taking for short-term prophylaxis)*

- Berinert® 500 / 1500
- Berinert® 2000 / 3000 (SC)
- Cinryze®
- Ruconest®
- Firazyr®
- Takhzyro®
- Androgens (e.g. danazol, stanozolol, oxandrolone)
- Tranexamic acid (e.g., Exacyl®)
- None of the above

*** Question C01.2:** Are you taking any other HAE medications other than those listed above?

Yes 🡪 Continue to **question C01.3**

No 🡪 Continue to **question C02**

**Question C01.3:** Please list here the other medications you are taking for prophylaxis and / or treating attacks *(Please only state one medication per line. For more than 2 medications, please indicate the most two frequent ones)*

| Medication 1 |  |
| --- | --- |
|  | |
| Medication 2 |  |

**Question C02:** Approximately how long have you been taking these medications? *(Please enter the year)*

***Products displayed to participants included all products selected in C01.1a and medications entered in question C01.3***

|  | Short-term prophylaxis | | | Long-term prophylaxis | | | Attack medication | | |
| --- | --- | --- | --- | --- | --- | --- | --- | --- | --- |
| 1. Selected medication |  |  |  |  |  |  |  |  |  |
|  | | | | | | | | | |
| 2. Selected medication |  |  |  |  |  |  |  |  |  |
|  | | | | | | | | | |
| 3. Selected medication |  |  |  |  |  |  |  |  |  |

………

**Question C03.1:** Have you switched one or more HAE medications in the past 12 months? *(Please check)*

- Yes 🡪 Continue to **question C03.2**
- No 🡪 Continue to **question C04**

**Question C03.2:** For what reason(s) did you switch the HAE medication? *(Please specify)*

**Question C04:** How satisfied are you with your current medication? (*Please check*)

***Products displayed to participants included all products selected in questions C01.1a-C01.1c and medications entered in question C01.3***

|  | Very satisfied | Quite satisfied | Somewhat satisfied | Somewhat dissatisfied | Quite dissatisfied | Very dissatisfied |
| --- | --- | --- | --- | --- | --- | --- |
| 1. Selected medication | 🌕 | 🌕 | 🌕 | 🌕 | 🌕 | 🌕 |
| 2. Selected medication | 🌕 | 🌕 | 🌕 | 🌕 | 🌕 | 🌕 |
| 3. Selected medication | 🌕 | 🌕 | 🌕 | 🌕 | 🌕 | 🌕 |
| ……. |  |  |  |  |  |  |

***The following question and table were displayed for all products selected in questions C01.1a - C01.1c and for medications entered in question C01.3***

**Question C05: How would you rate "***Name selected medications*" **in terms of the following?** *(Please check)*

|  | Applies fully | Applies | Somewhat applies | Somewhat does not apply | Does not apply | Does not apply at all |
| --- | --- | --- | --- | --- | --- | --- |
| Very effective | 🌕 | 🌕 | 🌕 | 🌕 | 🌕 | 🌕 |
| Very fast acting  *(only show if* ***attack treatment*** *is selected)* | 🌕 | 🌕 | 🌕 | 🌕 | 🌕 | 🌕 |
| Too frequent use / injection  *(only show if* ***long-term prophylaxis*** *is selected)* | 🌕 | 🌕 | 🌕 | 🌕 | 🌕 | 🌕 |
| Very easy to use | 🌕 | 🌕 | 🌕 | 🌕 | 🌕 | 🌕 |
| Complex documentation of intake | 🌕 | 🌕 | 🌕 | 🌕 | 🌕 | 🌕 |
| Shelf-life is too short | 🌕 | 🌕 | 🌕 | 🌕 | 🌕 | 🌕 |
| Very well tolerated / no side effects | 🌕 | 🌕 | 🌕 | 🌕 | 🌕 | 🌕 |
| Cooling is a big problem | 🌕 | 🌕 | 🌕 | 🌕 | 🌕 | 🌕 |
| Requires little time | 🌕 | 🌕 | 🌕 | 🌕 | 🌕 | 🌕 |
| Puncture during the injection is very uncomfortable | 🌕 | 🌕 | 🌕 | 🌕 | 🌕 | 🌕 |
| High availability / high delivery capacity | 🌕 | 🌕 | 🌕 | 🌕 | 🌕 | 🌕 |
| Very high volume / pressure sensation during the injection | 🌕 | 🌕 | 🌕 | 🌕 | 🌕 | 🌕 |

**Question C06:** With which physician(s) are you under regular care **due to HAE**? *(Please check – multiple answers are possible)*

- GP
- Dermatologist
- Allergist
- Pediatrician
- Gastroenterologist
- Pulmonologist
- Otolaryngologist
- HAE Centre
- Clinic / Hospital (no HAE centre)
- Other, please specify:

***If participants selected "HAE Centre" or "Clinic/Hospital", they were instructed to continue to questions C06A- C06D. Otherwise, participants were instructed to continue to C07***

**Question C06A:** Approximately how far are you from the HAE centre or clinic? *(Please check – only one entry possible)*

- Less than 50 km
- Between 50 and 200 km
- More than 200 km

**Question C06B:** How do you usually come to the centre or clinic? *(Please check – only one entry possible)*

- I drive a car to the centre myself
- I am being driven by car to the centre
- I travel by train / public transport
- Other, please specify:

**Question C06D:** How much do you think is your personal effort in the care at the centre or in the clinic?

|  | Very high | High | Rather high | Rather low | Low | Very low |
| --- | --- | --- | --- | --- | --- | --- |
| Scheduling | 🌕 | 🌕 | 🌕 | 🌕 | 🌕 | 🌕 |
| Appointment preparation (request referral, insured card) | 🌕 | 🌕 | 🌕 | 🌕 | 🌕 | 🌕 |
| Travel to and from | 🌕 | 🌕 | 🌕 | 🌕 | 🌕 | 🌕 |
| Waiting time during an appointment at the centre / clinic | 🌕 | 🌕 | 🌕 | 🌕 | 🌕 | 🌕 |
| Receipt of a prescription | 🌕 | 🌕 | 🌕 | 🌕 | 🌕 | 🌕 |

**Question C07:** Which doctor usually writes you the prescription for the HAE treatment? *(Please check – only one entry possible)*

- Doctor at the centre or clinic 🡪 Continue to **question C8**
- Primary care physician 🡪 Continue to **question C8**
- Other physician 🡪 Continue to **question C8**
- I have not received a prescription in the last two years 🡪 Continue to **Part D**

**Question C8:** How did you receive your last prescription for your medication(s) for the treatment of HAE? *(Please check – only one entry possible)*

- I have filled the prescription in a pharmacy
- I have received the medication(s) through an internet or mail-order pharmacy
- I have received the medication(s) through a nursing or patient service (e.g., homecare)
- Other, please specify:

**Question C9:** What is usually the amount you have to pay yourself when filling a prescription for your HAE medication? *(Please enter the amount in euros and the number of vials for which you pay the amount at the pharmacy)*

| I pay (euros) |  |
| --- | --- |
|  | |
| for the following number of vials: |  |

**Question C10:** There have been problems with the delivery of the medication over the last few years. What impact did the supply issue have on you personally? *(Please check – multiple answers are possible)*

- None
- Had to contact / visit the doctor
- I was prescribed another medication
- I was afraid that I could not be adequately treated
- I stocked up to be more independent in case of future supply issues
- After the supply issue was resolved, my doctor prescribed the medication again
- Other, please specify:

**Section D: Prophylaxis**

***If a product was selected in question C01.1b, question block D2 was presented to participants; otherwise, question block D1 was presented to participants***

***Section D: Start question block D2 (only patients currently using long-term prophylaxis)***

**Question D2-01:** Approximately when did you start using long-term prophylaxis? (*Please enter month and year – if month not known, please enter 00)*

| Month |  |  | Year |  |
| --- | --- | --- | --- | --- |

**Question D2-02:** How satisfied are you with your current long-term prophylaxis? *(Please tick)*

- Very satisfied
- Satisfied
- Somewhat satisfied
- Somewhat less satisfied
- Dissatisfied
- Not satisfied at all

**Question D2-03:** To what extent has current long-term prophylaxis improved your quality of life? *(Please check)*

- Very significantly
- Significantly
- Somewhat significantly
- Somewhat less significantly
- Less significantly
- Not at all

**Question D2-04:** Based on your experience with long-term prophylaxis, would you generally also recommend long-term prophylaxis to other HAE patients? *(Please check)*

- I would definitely recommend
- I would most likely recommend
- I would probably recommend
- I would probably not recommend
- I would most likely not recommend
- I would definitely not recommend

**Question D2-05:** What benefits of long-term prophylaxis do you see? *(Please check – multiple answers are possible)*

- Reduction in frequency of swelling attacks
- Reduction in the severity of swelling attacks
- Improvement in quality of life
- Reduction of the worry about swelling attacks
- Increased flexibility in work and personal life
- Do not see any benefits
- Other benefits *(please specify):*

**Question D2-06:** Do you also see disadvantages of long-term prophylaxis? *(Please check – multiple answers are possible)*

- Efficacy not sufficient
- Attacks can still occur
- Effort for preparation of the application (e.g., procurement / storage of the medication)
- Effort in application / cumbersome in application
- Regular use is often forgotten
- Tolerability issues / possible side effects
- Is uncomfortable to use
- Effort for documentation
- Do not see any disadvantages
- Other disadvantages *(please specify):*

**Question D2-07:** How much did the long-term prophylaxis reduce the frequency of your HAE attacks? *(Please check)*

- Very significantly
- Significantly
- Somewhat significantly
- Somewhat less significantly
- Less significantly
- Not at all
- Difficult to say / don't know

**Question D2-08:** And on average, how significantly did the long-term prophylaxis reduce the severity of your remaining HAE attacks? *(Please check)*

- Very significantly
- Significantly
- Somewhat significantly
- Somewhat less significantly
- Less significantly
- Not at all
- Difficult to say / don't know

**Question D2-09:** How do you feel about the time effort required for the preparation and application (preparation, administration, documentation) of the long-term prophylaxis? *(Please check)*

- Very high
- High
- Somewhat high
- Somewhat less high
- Low
- Very low

*** Question D02-10:** How often do you use the long-term prophylaxis? *(Please select the number of applications and the associated time unit – daily, weekly, monthly)*

| 🌕 1 time | 🌕 2 times | 🌕 3 times | 🌕 4 times | 🌕 5 times | 🌕 6 times | 🌕 7 times |
| --- | --- | --- | --- | --- | --- | --- |
| **per** |  |  |  |  |  |  |
| 🌕 day | 🌕 week | 🌕 month |  |  |  |  |

**Question D2-11:** Does it happen that you also forget or do not use the long-term prophylaxis once in a while? *(Please check, only one selection is possible)*

- Yes 🡪 Continue to **question D2-11.2**
- No 🡪 Continue to **question D02-12**

**Question D2-11.2:** Approximately how often does it happen that you forget the prophylaxis? *(Please check, only one selection is possible)*

- Several times a month
- 1x per month
- 1x per quarter
- 1-2x per year
- Less frequently than 1x per year
- Difficult to say / don't know

*** Question D2-12:** Assuming that long-term prophylaxis is available as a tablet which must be taken once a day and has a similar effect and tolerability as already available prophylaxis medications, how great is your interest in such long-term prophylaxis? (*Please check*)

- Very strong interest
- Strong interest
- Moderately strong interest
- Moderately weak interest
- Weak interest
- No interest at all
- Don't know

***End of section D: Question block D2 (only patients currently using long-term prophylaxis)***

🡪 Continue to **question D2-13** (page 19)

***Section D: Start question block D1 (only patients not currently using long-term prophylaxis)***

*** Question D1-01:** Have you ever used long-term prophylaxis before? *(Please check, only one selection is possible)*

- No 🡪 Continue to **question D1-01A**
- Yes 🡪 Continue to **question D1-01B**

**Question D01-1A**: What are the main reasons you decided against long-term prophylaxis? *For each statement on the list, please check how well it applies to you:*

|  | Applies fully | Applies | Somewhat applies | Somewhat does not apply | Does not apply | Does not apply at all |
| --- | --- | --- | --- | --- | --- | --- |
| I have attacks only very rarely | 🌕 | 🌕 | 🌕 | 🌕 | 🌕 | 🌕 |
| My attacks are not particularly severe | 🌕 | 🌕 | 🌕 | 🌕 | 🌕 | 🌕 |
| The documentation of the prophylaxis is too time-consuming for me | 🌕 | 🌕 | 🌕 | 🌕 | 🌕 | 🌕 |
| I am not convinced about the efficacy of prophylaxis | 🌕 | 🌕 | 🌕 | 🌕 | 🌕 | 🌕 |
| My attack medication is completely sufficient for me | 🌕 | 🌕 | 🌕 | 🌕 | 🌕 | 🌕 |
| I don't want to be constantly reminded of HAE through the prophylaxis | 🌕 | 🌕 | 🌕 | 🌕 | 🌕 | 🌕 |
| My doctor has advised me against prophylaxis | 🌕 | 🌕 | 🌕 | 🌕 | 🌕 | 🌕 |
| The use of prophylaxis is too complex for me | 🌕 | 🌕 | 🌕 | 🌕 | 🌕 | 🌕 |
| I am not convinced about the pharmaceutical form of the prophylaxis (e.g., infusion, syringe, etc) | 🌕 | 🌕 | 🌕 | 🌕 | 🌕 | 🌕 |

🡪 Continue to **question D1-02**

**Question D1-01B**: What were the main reasons you discontinued the long-term prophylaxis? *For each statement on the list, please state how well it applies to you:*

|  | Applies fully | Applies | Somewhat applies | Somewhat does not apply | Does not apply | Does not apply at all |
| --- | --- | --- | --- | --- | --- | --- |
| I have attacks only very rarely | 🌕 | 🌕 | 🌕 | 🌕 | 🌕 | 🌕 |
| My attacks are not particularly severe | 🌕 | 🌕 | 🌕 | 🌕 | 🌕 | 🌕 |
| The documentation of the prophylaxis was too time-consuming for me | 🌕 | 🌕 | 🌕 | 🌕 | 🌕 | 🌕 |
| I was not convinced about the efficacy of prophylaxis | 🌕 | 🌕 | 🌕 | 🌕 | 🌕 | 🌕 |
| My attack medication is completely sufficient for me | 🌕 | 🌕 | 🌕 | 🌕 | 🌕 | 🌕 |
| I don't want to be constantly reminded of HAE through the prophylaxis | 🌕 | 🌕 | 🌕 | 🌕 | 🌕 | 🌕 |
| The use of prophylaxis was too complex for me | 🌕 | 🌕 | 🌕 | 🌕 | 🌕 | 🌕 |
| My doctor has advised me against prophylaxis | 🌕 | 🌕 | 🌕 | 🌕 | 🌕 | 🌕 |
| I did not use the prophylaxis regularly anyway | 🌕 | 🌕 | 🌕 | 🌕 | 🌕 | 🌕 |
| I did not tolerate the prophylaxis well | 🌕 | 🌕 | 🌕 | 🌕 | 🌕 | 🌕 |

🡪 Continue to **question D1-02**

**Question D1-02:** In your opinion, what speaks in favor of long-term prophylaxis? *(Please check - multiple answers are possible)*

- Reduction in the frequency of swelling attacks
- Reduction in the severity of swelling attacks
- Improvement in quality of life
- Reduced concerns about swelling attacks
- Increased flexibility in work and personal life
- Do not see any benefits
- Other benefits *(please specify):*

**Question D1-03:** In your opinion, what speaks against long-term prophylaxis? (*Please check – multiple answers are possible*)

- Efficacy is not sufficient
- Attacks can still occur
- Effort to preparation treatment administration (e.g., procurement / storage of the medication)
- Effort for treatment administration / cumbersome administration
- Effort for the documentation of treatment administration
- Regular use is often forgotten
- Tolerability issues / possible side effects
- Discomfort with use
- Do not see any disadvantages
- Other disadvantages *(please specify):*

*** Question D1-04:** Would an easy-to-use long-term prophylaxis be a reason for you to reconsider your decision? *(Please check)*

- Yes
- No
- Don't know

*** Question D1-05:** Assuming that long-term prophylaxis is available as a tablet which must be taken once a day and which has a similar effect and tolerability as already available prophylaxis medications, how great is your interest in such long-term prophylaxis? *(Please check)*

- Very strong interest
- Strong interest
- Moderately strong interest
- Moderately weak interest
- Weak interest
- No interest at all
- Don't know

***End of question block D1 (only patients not*** ***currently using long-term prophylaxis)***

🡪 Continue to **question D2-13**

***The following questions will be asked to all patients:***

**Question D2-13:** How would you rate the following statements about a tablet as long-term prophylaxis that must be taken once a day? *(Please check)*

|  | Applies fully | Applies | Somewhat applies | Somewhat does not apply | Does not apply | Does not apply at all |
| --- | --- | --- | --- | --- | --- | --- |
| Is very easy to use | 🌕 | 🌕 | 🌕 | 🌕 | 🌕 | 🌕 |
| Does not require any time effort | 🌕 | 🌕 | 🌕 | 🌕 | 🌕 | 🌕 |
| Can be easily forgotten at the start of therapy | 🌕 | 🌕 | 🌕 | 🌕 | 🌕 | 🌕 |
| Is easy to integrate into the daily routine | 🌕 | 🌕 | 🌕 | 🌕 | 🌕 | 🌕 |
| Is no longer forgotten with long-term use, since it becomes routine | 🌕 | 🌕 | 🌕 | 🌕 | 🌕 | 🌕 |
| A recommendation by the doctor is very important | 🌕 | 🌕 | 🌕 | 🌕 | 🌕 | 🌕 |
| A recommendation by other HAE patients is very important | 🌕 | 🌕 | 🌕 | 🌕 | 🌕 | 🌕 |
| A patient diary is very beneficial to document the intake | 🌕 | 🌕 | 🌕 | 🌕 | 🌕 | 🌕 |
| Storage is not an issue | 🌕 | 🌕 | 🌕 | 🌕 | 🌕 | 🌕 |
| Having an aid to remind you to take it is very important (e.g., iPhone/smartphone app) | 🌕 | 🌕 | 🌕 | 🌕 | 🌕 | 🌕 |
| When traveling, a tablet is much more convenient than an injection | 🌕 | 🌕 | 🌕 | 🌕 | 🌕 | 🌕 |

**Question D02-14:** Would you try this type of prophylaxis? *(Please select one of the following answers)*

- Definitely
- Very likely
- Likely
- Less likely
- Unlikely
- Under no circumstances
- Don't know

***Section E: Service & Support***

*** Question E01:** Various services are offered for HAE patients; additional services are possible. Please rate how useful the following services are **for you**. *(Please check)*

|  | Very useful | Useful | Somewhat useful | Somewhat less useful | Less useful | Not at all useful | Don't know |
| --- | --- | --- | --- | --- | --- | --- | --- |
| Patient diary / swelling calendar | 🌕 | 🌕 | 🌕 | 🌕 | 🌕 | 🌕 | 🌕 |
| Emergency ID card | 🌕 | 🌕 | 🌕 | 🌕 | 🌕 | 🌕 | 🌕 |
| Educational materials on traveling | 🌕 | 🌕 | 🌕 | 🌕 | 🌕 | 🌕 | 🌕 |
| HAE expert search on the internet | 🌕 | 🌕 | 🌕 | 🌕 | 🌕 | 🌕 | 🌕 |
| Treatment guide | 🌕 | 🌕 | 🌕 | 🌕 | 🌕 | 🌕 | 🌕 |
| Information about the disease in general | 🌕 | 🌕 | 🌕 | 🌕 | 🌕 | 🌕 | 🌕 |
| Patient experience / patient stories | 🌕 | 🌕 | 🌕 | 🌕 | 🌕 | 🌕 | 🌕 |
| Caregivers / nurses coming to your home for injection training (Homecare / Care service) | 🌕 | 🌕 | 🌕 | 🌕 | 🌕 | 🌕 | 🌕 |
| Medication delivery service | 🌕 | 🌕 | 🌕 | 🌕 | 🌕 | 🌕 | 🌕 |
| Doctor’s video / telephone clinic | 🌕 | 🌕 | 🌕 | 🌕 | 🌕 | 🌕 | 🌕 |
| Reminder of doctor's appointments | 🌕 | 🌕 | 🌕 | 🌕 | 🌕 | 🌕 | 🌕 |

**Question E02:** Which of the following services / support materials for HAE patients have you used in the past 12 months? *(Please check – multiple answers are possible)*

- Patient diary / swelling calendar
- Emergency ID card
- Educational materials on traveling
- Educational materials on pregnancy
- HAE expert search on the internet
- Treatment guide
- Information about the disease in general
- Patient experience / patient stories
- Caregivers / nurses coming to your home for injection training (Homecare / Care service)
- Medication delivery service
- Doctor’s video / telephone clinic
- *Other, please specify:*

*** Question E3:** Would other services be helpful for you? *(Please check / enter)*

- No
- Yes, the following services would still be helpful *(please enter):*

***Section F: Statistics***

*** Question F1:** In which country do you have your primary residence? *(Please check)*

- Germany 🡪 Please also indicate the state:

| 🌕 Baden-Württemberg | 🌕 Bavaria | 🌕 Berlin | 🌕 Brandenburg |
| --- | --- | --- | --- |
| 🌕 Bremen | 🌕 Hamburg | 🌕 Hessen | 🌕 Mecklenburg-Western Pomerania |
| 🌕 Lower Saxony | 🌕 North Rhine-Westphalia | 🌕 Rhineland-Palatinate | 🌕 Saarland |
| 🌕 Saxony | 🌕 Saxony-Anhalt | 🌕 Schleswig-Holstein | 🌕 Thuringia |

- Austria
- Switzerland

*** Question F02:** How many inhabitants are there where you reside? *(Please check)*

- Less than 5,000 inhabitants
- 5,000 – 10,000 inhabitants
- 10,001 – 50,000 inhabitants
- 50,001 – 100,000 inhabitants
- 100,001 – 250,000 inhabitants
- 250,001 – 500,000 inhabitants
- More than 500,000 inhabitants

*** Question F03:** What is your age group? *(Please check)*

- Under 15 years
- 15 - 17 years
- 18 - 29 years
- 30 - 39 years
- 40 - 49 years
- 50 - 59 years
- 60 - 69 years
- 70 years and older
- No statement

**Question F04**: Please indicate your gender: *(Please check)*

- Male
- Female
- Diverse
- No statement

**Question F05**: What type of health insurance do you have? *(Please check)*

- Statutory health insurance
- Private health insurance
- Other health insurance
- No statement

If you would like to provide additional comments about HAE or the survey, you can enter them here:

**Thank you for your participation!**
